# Depth-based registration of 3D preoperative models to intraoperative patient anatomy using the HoloLens 2

**DOI:** 10.1007/s11548-025-03328-x

**Published:** 2025-03-14

**Authors:** Enzo Kerkhof, Abdullah Thabit, Mohamed Benmahdjoub, Pierre Ambrosini, Tessa van Ginhoven, Eppo B. Wolvius, Theo van Walsum

**Affiliations:** 1 Department of Radiology & Nuclear Medicine, Biomedical Imaging Group Rotterdam, Erasmus MC, Rotterdam, The Netherlands; 2 Department of Surgical Oncology and Gastrointestinal Surgery, Erasmus MC Cancer Institute, Rotterdam, The Netherlands; 3 Department of Oral and Maxillofacial Surgery, Erasmus MC, Rotterdam, The Netherlands; 4 Department of Surgical Oncology, Netherlands Cancer Institute, 1066 CX Amsterdam, The Netherlands

**Keywords:** Image-guided surgery, Augmented reality, Head-mounted displays, HoloLens 2, Depth-based registration, Image-to-patient registration

## Abstract

****Purpose**:**

In augmented reality (AR) surgical navigation, a registration step is required to align the preoperative data with the patient. This work investigates the use of the depth sensor of HoloLens 2 for registration in surgical navigation.

****Methods**:**

An AR depth-based registration framework was developed. The framework aligns preoperative and intraoperative point clouds and overlays the preoperative model on the patient. For evaluation, three experiments were conducted. First, the accuracy of the HoloLens’s depth sensor was evaluated for both Long-Throw (LT) and Articulated Hand Tracking (AHAT) modes. Second, the overall registration accuracy was assessed with different alignment approaches. The accuracy and success rate of each approach were evaluated. Finally, a qualitative assessment of the framework was performed on various objects.

****Results**:**

The depth accuracy experiment showed mean overestimation errors of 5.7 mm for AHAT and 9.0 mm for LT. For the overall alignment, the mean translation errors of the different methods ranged from 12.5 to 17.0 mm, while rotation errors ranged from 0.9 to 1.1 degrees.

****Conclusion**:**

The results show that the depth sensor on the HoloLens 2 can be used for image-to-patient alignment with 1–2 cm accuracy and within 4 s, indicating that with further improvement in the accuracy, this approach can offer a convenient alternative to other time-consuming marker-based approaches. This work provides a generic marker-less registration framework using the depth sensor of the HoloLens 2, with extensive analysis of the sensor’s reconstruction and registration accuracy. It supports advancing the research of marker-less registration in surgical navigation.

**Supplementary Information:**

The online version contains supplementary material available at 10.1007/s11548-025-03328-x.

## Introduction

Surgical procedures often require good comprehension of the patient anatomy and the ability to mentally map preoperative plans to the operating table. With technologies such as surgical navigation systems, surgeons can translate the preoperative plans to the operating table easily, therefore improving patient outcome [1]. The alignment of the preoperative imaging data to the patient anatomy, known as the image-to-patient registration, is a crucial step in surgical navigation.

Conventional navigation systems often use markers that are rigidly attached to the patient and a stylus or pointer to perform the image-to-patient registration. Marker-based registration is time-consuming and requires the attachment of reference markers to the patient, which can be invasive. Furthermore, conventional navigation systems are expensive and have a complex setup, which hampers their wide adaptation in many surgical applications [1].

With the development of AR head-mounted displays (HMDs), the accessibility of visualizing 3D preoperative models intraoperatively is increasing [2]. Compared to traditional navigation approaches, AR-based navigation can visualize the information directly on the patient, in the view of the surgeon, reducing hand–eye coordination issues as well as the switch of focus between the patient and the monitor. However, to navigate with AR HMDs the 3D models need to be registered to the intraoperative patient space [3]. Using a traditional navigation system for this registration task includes the previously mentioned cumbersome registration setup. Instead, using AR HMDs with marker-less registration has the potential of replacing the traditional registration approach with a more automated approach, using the device’s sensors. This may simplify surgical navigation, therefore enabling navigation for surgical applications that currently do not use traditional image-guided navigation systems due to the added complexity [2]. In marker-less registration, features from RGB images are used to directly estimate the pose of the target object and register the preoperative model [4]. The detection of features in the RGB image was investigated by von Atzigen et al., where implanted screw heads were detected to assist in rod bending in spine surgery [5]. Similarly, Benmahdjoub et al. proposed a proof of concept where surgical landmarks were detected as sparse features to register a phantom skull [6] using the HoloLens. Moreover, Doughty et al. proposed HMD-EgoPose, a CNN-based solution, which extracts features from the RGB feed and estimates the pose of the surgeon’s hands and the instrument being used [7].

One promising marker-less approach for addressing the registration challenge lies in leveraging the depth sensor of the HoloLens 2. The depth sensor acquires 3D data which can be used for registering preoperative models to the intraoperative patient space, eliminating the need for manual collection of registration points. By utilizing depth-based methods, it may become feasible to achieve fast, easy and automatic image-to-patient registration. Depth-based registration approaches have been proposed before in several studies [8–12]. Nonetheless, the current published research is still limited in numbers, often application specific and rarely translated to routine patient care.

The aim of this study is to evaluate the feasibility of using the depth sensor of the HoloLens 2 AR HMD for general depth-based image-to-patient registration and assess initialization approaches which have direct impact on the registration accuracy and system usability (nondisruptive to the clinical workflow). Compared to previous studies, which investigate the use of the HoloLens 1, focus on specific anatomical regions, assess only the registration accuracy, or focus on reconstruction methods ( [8, 9, 12–14]), we perform experiments that focus on assessing the accuracy, robustness, speed, and automation of different depth-based registration methods, assessing the depth estimation of the HoloLens 2, and provide a generalized depth registration framework implemented on the HoloLens 2, which can be fine-tuned to specific anatomical regions. Our study contributes to the broader goal of achieving precise and automated image-to-patient registration, thereby enhancing the efficiency and accessibility of image-guided surgery.

## Methods

In this section, the proposed AR depth-based registration framework is described, including the reconstruction of the source and target point clouds as well as the registration approaches for image-to-patient alignment.

### HoloLens 2 depth registration approach

**Fig. 1:**
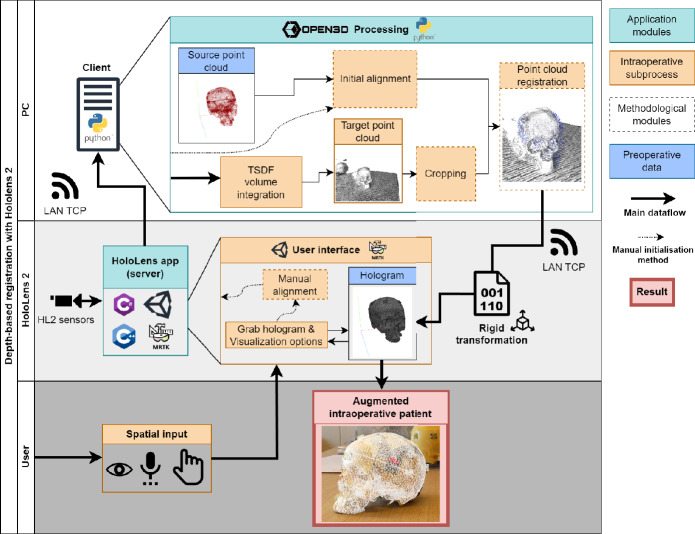
Depth-based registration framework: the workflow and interconnection between the HoloLens and the client PC

The depth registration framework consists of a HoloLens 2 (HL2) device and a PC connected to the same network. The HL2 provides a user interface that allows the user to manipulate the preoperative model and transmit its initial pose along with the captured frames of the depth sensor and user spatial input to the client PC. The PC processes the preoperative and intraoperative depth data to find the image-to-patient registration matrix and sends it back to the HL2. The HL2 then visualizes the preoperative model aligned on the target patient’s anatomy. This client–server communication is enabled using the HL2 sensor streaming (hl2ss) unity plugin [15], which leverages the capabilities of the Microsoft HL2 Research Mode to access raw sensor data [16]. Figure 1 provides an overview of the framework’s components, illustrating their interconnections and data flow.

### Preoperative source point cloud

**Fig. 2:**
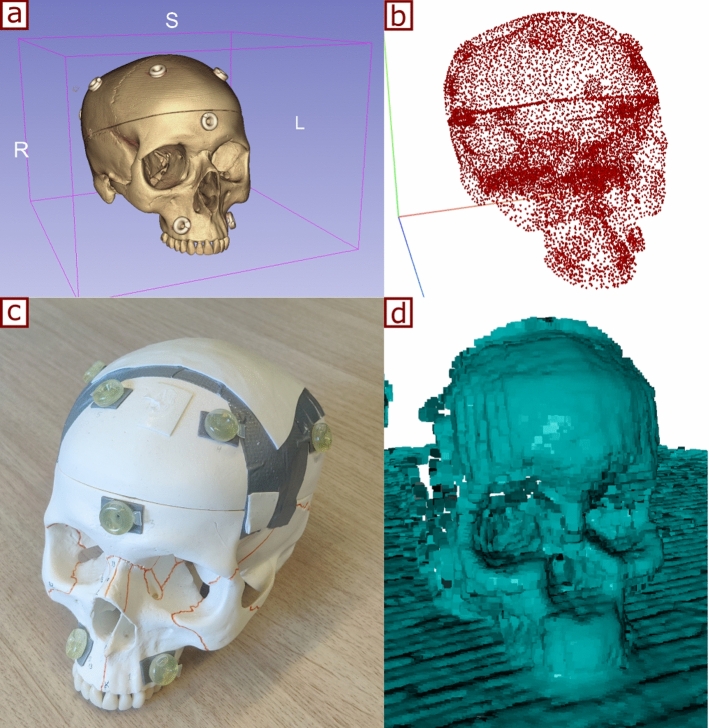
Reconstruction of source and target point clouds: **a** preoperative 3D model, **b** source point cloud, **c** target anatomy, **d** target point cloud

Preoperative imaging, such as CT or MRI, is commonly performed to assist surgeons in planning surgical procedures. However, for the imaging data to be directly superimposed on the patient during surgery, a non-volumetric representation of the preoperative image is needed. For that, the target anatomical structure was segmented from the CT image and a surface mesh model was generated using 3D Slicer (www.slicer.org). The vertices of the mesh model were then used as the source point cloud to be aligned with intraoperatively. Figure 2a and b shows the preoperative model and the source point cloud, respectively.

### Intraoperative target point cloud

To align the preoperative model with the patient anatomy during surgery, an intraoperative representation is required. This is achieved by capturing the surface of the patient’s anatomy using depth cameras. The Microsoft HL2 is equipped with a time-of-flight (ToF) depth sensor that operates in two modes: Articulated Hand Tracking (AHAT), which operates at a high frequency of 45 frames per second (FPS), has an image resolution of 512x512 and is used for hand tracking, and the Long-Throw (LT) mode, which operates at a lower frequency of 1–5 FPS, has an image resolution of 320x288 and is used for spatial mapping [16]. This sensor captures depth information of the scene, enabling the reconstruction of the 3D environment.

As shown in Fig. 1, the client PC receives the depth frames and uses Truncated Signed Distance Function (TSDF) volume integration to reconstruct a 3D point cloud from multiple depth frames [17, 18]. For that, 50 depth frames are collected from different angles with the user moving around the region of interest during approximately 10 s. This allows for the creation of a more comprehensive 3D representation of the intraoperative scene. The 10-s duration was determined empirically, where it was sufficient to obtain multiple views to reconstruct the target object. A final cropping step, based on a geometry-fitting bounding box scaled by 1.1$-$1.5 times the size of the preoperative model, is used to crop the target point cloud (see Fig. 2d). This bounding box is centered around the initial alignment pose, and its size (1.1$-$1.5 times) was defined empirically.

### Image-to-patient alignment

After reconstructing the source point cloud of the preoperative model and the target point cloud of the intraoperative anatomical structure, the alignment between the two point clouds is established (see Fig. 1).

In our approach, we combine the Iterative Closest Point (ICP) algorithm with an initial registration approach which initializes the ICP based on an approximate alignment. For the initial registration approach, different manual and automated methods were evaluated (see Sect. 3.2). After the full alignment, the preoperative model can be visualized at the correct location superimposed on the target anatomy.

## Experiments & results

### Depth sensor accuracy

The accuracy of the depth sensor is important for accurate image-to-patient alignment, since the depth data serves as the intraoperative target for registration. This experiment aims to evaluate the accuracy of the depth frames captured by the AHAT and LT modes in reconstructing accurate point clouds while changing different factors, such as the distance to target objects, lighting conditions, and the HoloLens device used. To that end, the experiment evaluates the heights of different objects estimated by the depth sensor against their real heights.

**Fig. 3:**
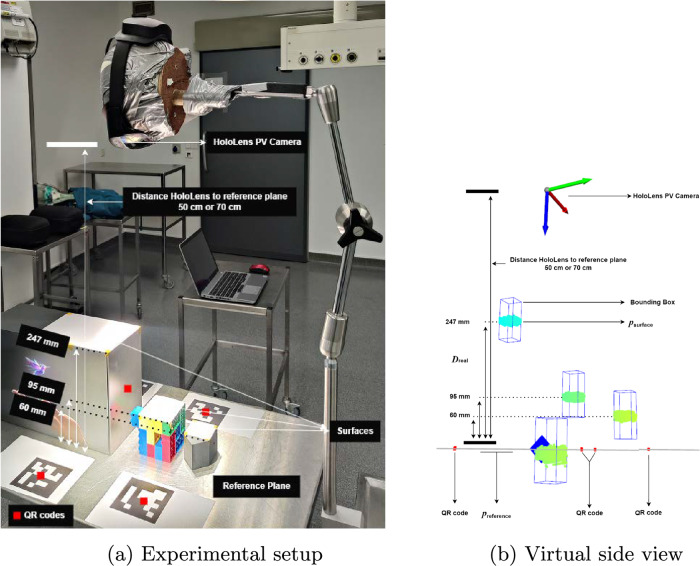
Experimental setup for measuring the accuracy of HL2 depth sensor

For the experimental setup, three objects with flat surfaces and of known dimensions were placed on a flat table, with the table itself functioning as a fourth surface (see Fig. 3a). The objects used in the experiment consisted of a 3D printed rectangular house-shaped model, a Lego cube, and a box, all covered with regular white printer paper to ensure similar surface material characteristics. The HoloLens was mounted on a stand and positioned to look perpendicularly to the target surfaces, similar to how a user would normally look at an object. To ensure the perpendicular view from the user perspective, four ArUco markers [19] with a width of 10 cm were printed on white papers and placed on the table surface. The four markers were located by the Personal Video (PV) camera of the HoloLens and were used to (1) establish a reference plane that estimated the distance of the table surface to the PV camera, and (2) ensure a 180±5 degrees (perpendicular view) between the reference plane’s normal vector and the PV camera’s forward axis. For ground- truth measurements, the plane established by the ArUco markers (representing the table surface) acted as a reference plane. Then, given the known dimensions of the objects, their height from the table surface served as the ground-truth height measurements.

During each experiment, the extrinsics of the HoloLens’s sensors were retrieved using the hl2ss plugin, and data were recorded for 30 s (poses of markers through the PV camera and depth frames through the depth sensor). For the PV camera, the reference plane constructed by the ArUco markers was transformed from the PV coordinate system to the device world space. For the depth sensor, depth frames were converted into point clouds and transformed to the device world space. All depth points were combined to form a single point cloud representing the entire 30-seconds acquisition. From this comprehensive point cloud, the flat surfaces of the target objects were manually selected and cropped using bounding boxes with dimensions of 3x3x8 cm, ensuring a consistent surface area for calculating the depth estimation error (see Fig. 3b). For the table surface, the cropping bounding box was positioned to collect depth points only from the white paper surfaces placed on the table, in order to maintain consistency with the surface material of the other target objects and to ensure the measurements will not be influenced by the material of the table. After that, the relative distances between all the points in the cropped bounding box and the reference plane were calculated (along the direction of the plane’s normal vector). For each target surface, the expected distance to the reference plane is known and should be 0 mm (table), 60 mm (house-shaped model), 95 mm (Lego cube), or 247 mm (box), corresponding to each of the target objects. The distance between the estimated position of the object’s surface ($p_{\text {surface}}$) and the position of the reference plane ($p_{\text {reference}}$) subtracted from the known object height ($D_{\text {real}}$) gives the error in depth estimation ($D_{\text {error}}$). The sign of this error indicates whether the depth is overestimated (positive) or underestimated (negative):

$$\begin{aligned}D_{\text {error}} = D_{\text {real}} - |\overrightarrow{p_{\text {surface}}p_{\text {reference}}}|\end{aligned}$$

To assess the effect of the operating room’s (OR) lighting conditions on the depth sensor accuracy compared to normal lighting conditions, the experiment was conducted twice: once in the lab with normal light and once in the OR with the OR light being on (Fig. 3a). Both experiments were repeated with two different HoloLens devices to evaluate the consistency across multiple devices.

**Fig. 4:**
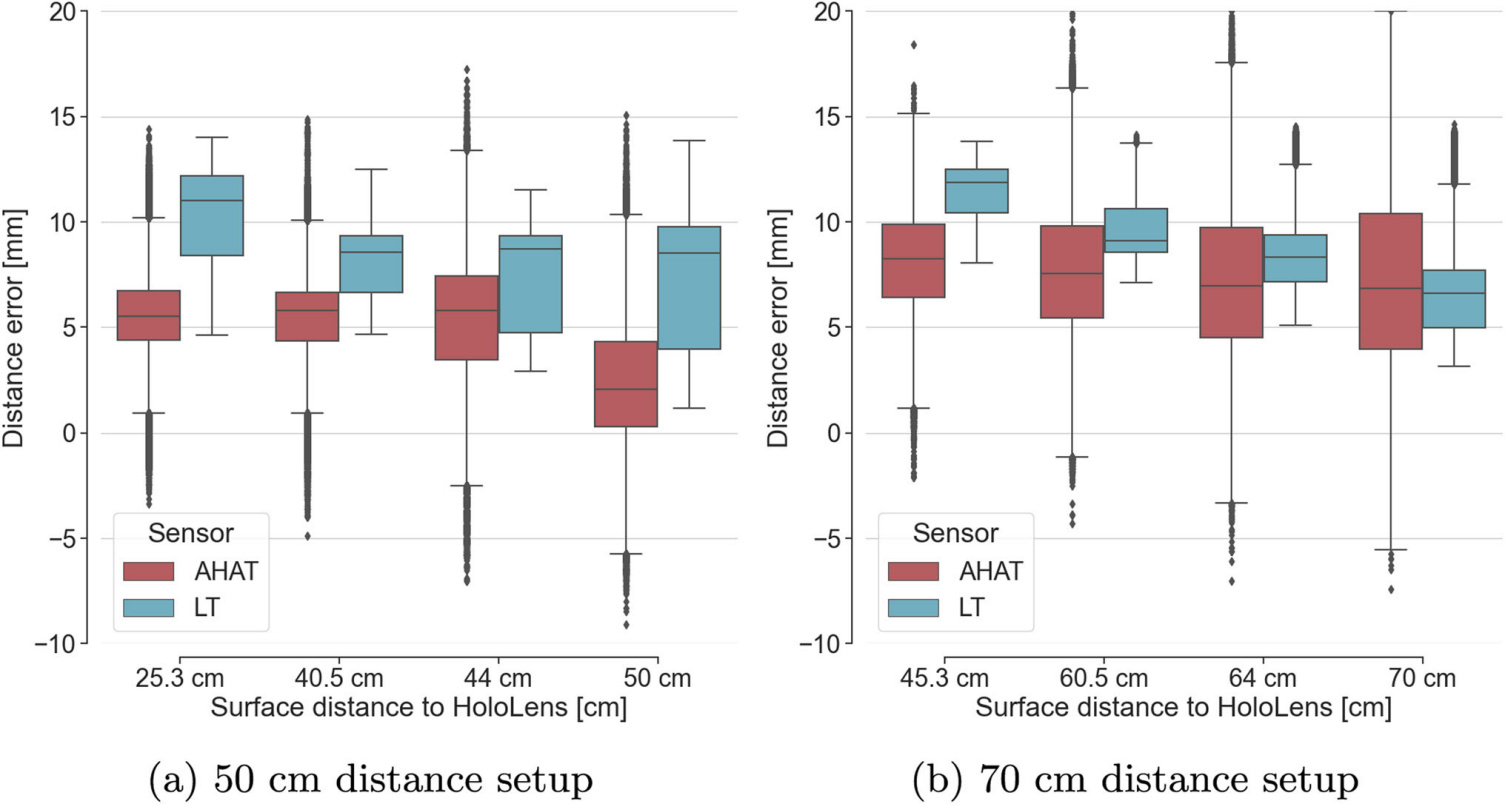
Depth estimation errors at different distances to the HL2 for the AHAT and LT mode

**Fig. 5:**
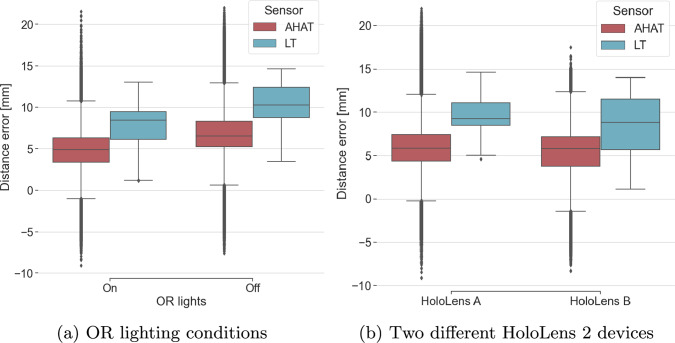
Impact of lighting conditions and device variability on depth estimation errors

Given the static setup of the experiment, it was assumed no drifting happened and all reported errors correspond to the depth estimation accuracy. This was confirmed by observing the position of the PV camera and ArUco markers in device world space across the 30-seconds acquisition, where the reported mean of standard deviations for all conducted experiments was lower than 0.3 mm. Table 1 shows the mean, minimum and maximum standard deviations of the position for both PV camera and ArUco markers across the acquisition time.

Figure 4 presents the depth estimation error with respect to the surface distance of the objects from the HL2, with the HL2 being placed in a perpendicular position facing the table reference plane at 50 and 70 cm distances. At both distances, the depth frames of AHAT and LT were recorded for 30 s.

From Fig. 4, we can see that both modes (AHAT and LT) exhibit a mean overestimation error for all surfaces. The LT mode generally shows a higher mean overestimation, although this difference decreases for surfaces located further than 50 cm. The standard deviation of the AHAT mode increases with the increase in the distance to the surface while the standard deviation of the LT decreases.

Figure 5 displays the depth estimation errors for all acquisitions categorized by lighting conditions and HoloLens device. The difference observed in mean depth estimation error between the two HoloLens devices for AHAT and LT mode was 0.74 mm and 1.34 mm, respectively, while for OR lighting conditions, a decrease in mean error for AHAT and LT mode of 1.95 mm and 2.40 mm with the OR lights on was observed compared to normal lighting conditions in the laboratory.

**Table 1:** Position standard deviation for the PV camera and ArUco markers over the 30-seconds acquisition time

Position standard deviation	x [mm] mean/min/max	y [mm] mean/min/max	z [mm] mean/min/max
ArUco markers (in world space)	0.18/0.05/0.57	0.36/0.11/0.75	0.19/0.06/0.73
PV camera (in world space)	0.17/0.04/0.59	0.17/0.05/0.67	0.21/0.03/0.53
ArUco markers (in PV coordinates)	0.09/0.05/0.14	0.27/0.09/0.53	0.08/0.04/0.14

### Depth registration accuracy

**Fig. 6:**
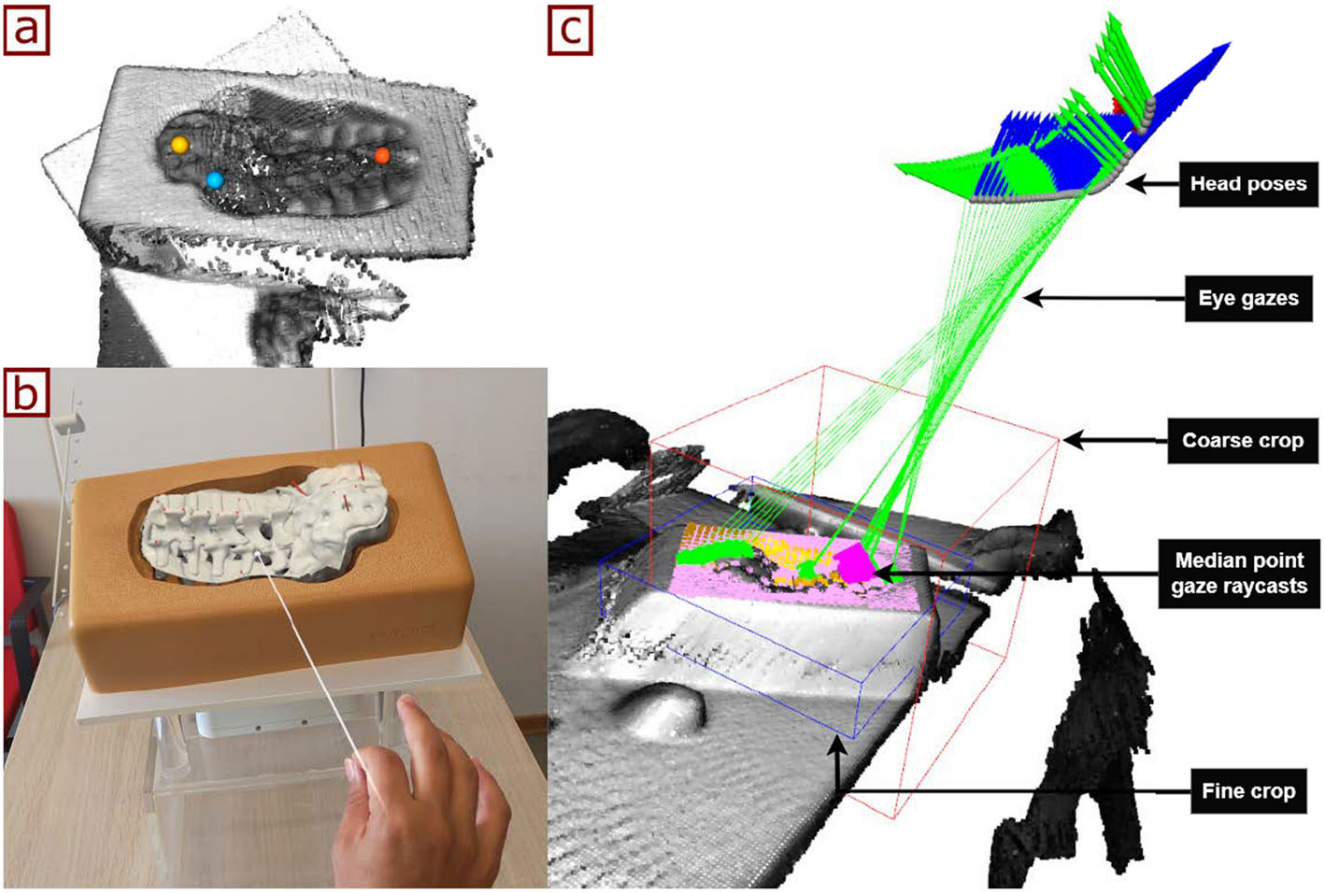
Registration approaches: **a** 3-point-picking, **b** Manual alignment, **c** Eye and Eye RANSAC

This experiment evaluates four different registration methods that were used to perform an initial alignment with the preoperative model before further refinement with ICP (see Sect. 2.4). The evaluated initial alignment methods are detailed below:

1. **3P (3-Point Picking)**: After acquiring the depth frames with the HoloLens, the user has to interact with the client PC monitor, where both the target point cloud and the source point cloud were displayed consecutively. The user has to manually select three corresponding points between the point clouds to initialize the ICP (see Fig. 6a).
2. **Manual**: The user tries to roughly align the preoperative model through manual manipulation in the HL2 app, where the approximate pose is then used to crop (as mentioned in Sect. 2.3) the target point cloud and initialize the ICP algorithm (see Fig. 6b).
3. **Eye**: The eye gaze spatial input of the user is collected while observing several sides of the target object up until the user gives a voice command to start the alignment process. The median position of last 100 spatial input data points, captured at a frequency of 30 Hz, was then used to translate the preoperative model, crop the target point cloud, and initialize the ICP algorithm (see Fig. 6c).
4. **Eye RANSAC**: Similar to the Eye method above, the preoperative model is translated to the median eye gaze input. After that, a fast global registration followed by RANSAC registration of Open3D was applied, respectively, for coarse alignment of cropped target point cloud surrounding the median eye gaze point [18]. The resulting pose from the RANSAC registration was used to crop the target point cloud a second time and initialize the ICP algorithm.

To assess the performance of the registration methods, a ground-truth alignment was established using the multi-modal system described in Benmahdjoub et al. [20], which has shown to have a target registration error (TRE) of 2 mm in [21]. The multi-modal system couples the HoloLens to an electromagnetic (EM) NDI tracking system using a calibrated Vuforia marker, where the registration of the preoperative model is performed using the EM pointer and transformed to the HoloLens coordinates through the calibrated marker.

The difference in the preoperative model’s final pose between the ground truth and the evaluated registration method was used to calculate the rotational and translational errors. To ensure correct error analysis, failed registration attempts, that happened when the ICP step failed to converge to a correct alignment, were excluded. A rotational error higher than 10 degrees was determined (empirically) as the exclusion criterion, where rotational misalignment was clearly visible. Failed registrations were excluded from the mean rotational and translational error analysis. Moreover, the time required to get the final pose of the preoperative model for each method was recorded.

**Fig. 7:**
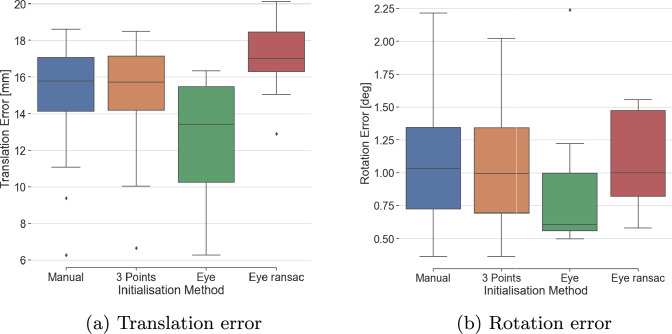
Registration errors of the depth-based methods

For this experiment, a spine lumbar vertebrae phantom model (Sawbones, Vashon Island, WA, USA) was used (see Fig. 6). For each method, 32 registration attempts were made with the spine model placed in 32 different orientations. The HoloLens had to remain within a clinically relevant range (an arm distance) to the spine model, and the rotation of the spine phantom was manually varied given that clinically the target structure can be oriented in multiple ways. The quantitative results for each registration method are summarized in Table 2. The mean translation errors of the four initialization methods ranged between 12.5 and 17.0 mm and rotation errors between 0.9 and 1.1 degrees. Box plots of the translation error (TE) and rotation error (RE) for each method are shown in Fig. 7. Methods 3P and Manual consistently provided successful registration (100%). However, the Eye and Eye RANSAC initialization methods succeeded in only 22% and 34% of the times, respectively. The time presented in Table 2 indicates the PC processing time for each method, with 3P being the slowest alignment approach with a mean time of 26.4 s. The other three methods all achieved a registration time of less than 4 s after giving the alignment voice command.

**Table 2:** Results for 32 registrations, TE is translation error, RE is rotation error

Method	Fail	TE [mm]	RE [deg]	Time [s]
Manual	0	15.14 ± 2.62	1.09 ± 0.52	2.15 ± 0.17
3P	0	15.19 ± 2.57	1.07 ± 0.49	26.44 ± 3.99
Eye	25*	12.50 ± 3.74	0.92 ± 0.63	3.12 ± 0.26
Eye RANSAC	21*	17.03 ± 2.00	1.11 ± 0.37	3.27 ± 0.36

*Failed registrations were excluded from the mean rotational and translational error analysis

### Qualitative assessment

**Fig. 8:**
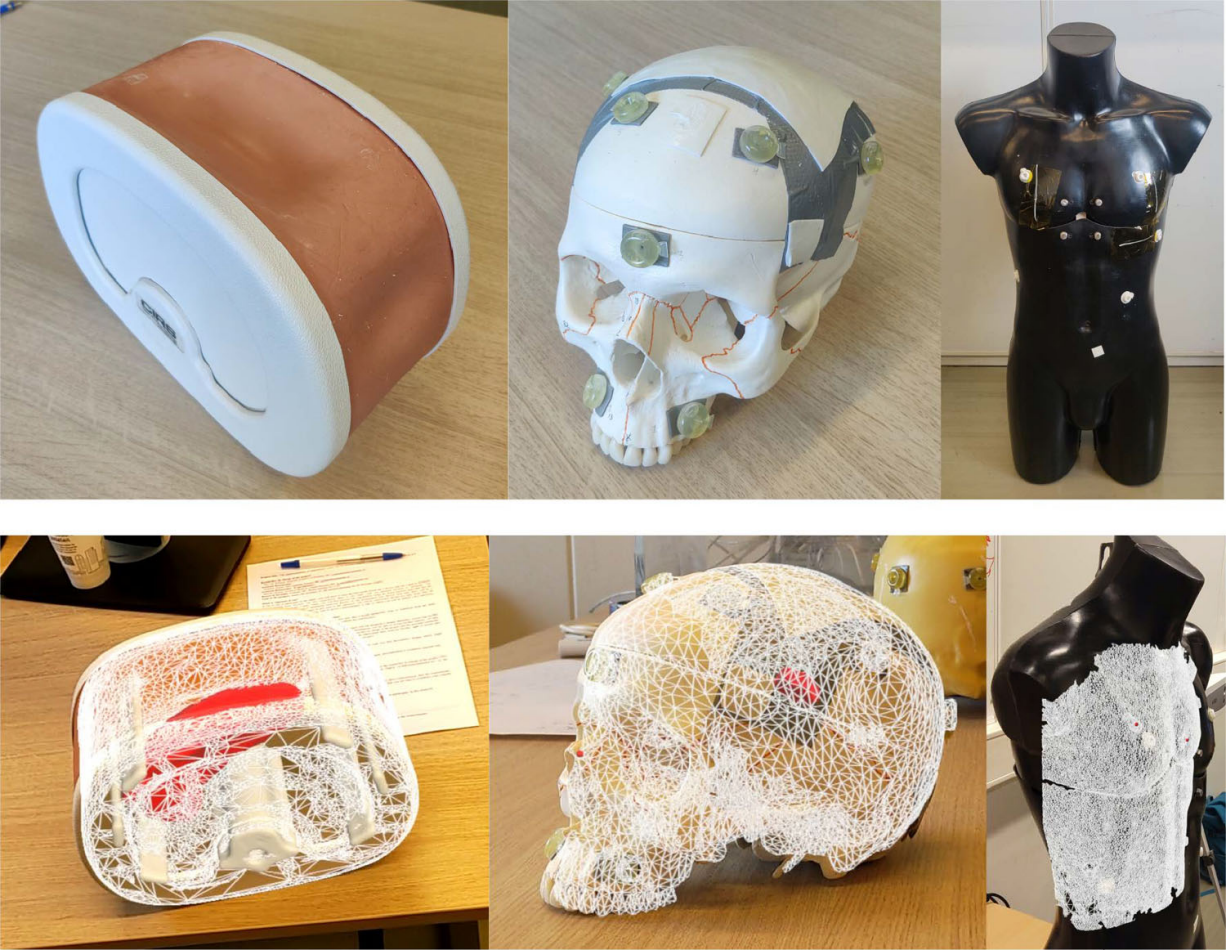
Objects used for the qualitative assessment along with their preoperative models aligned (left to right: abdominal phantom, skull phantom, mannequin)

**Fig. 9:**
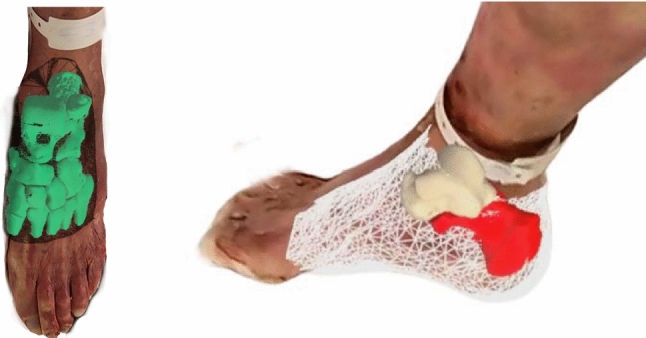
Cadaver feet with preoperative model overlays after depth-based registration

Based on the results of the registration method experiment, the Manual method was chosen for further qualitative analysis in this experiment, focusing on its applicability to a range of objects with varying surface properties, geometries, and material types. The objects selected for the qualitative assessment are shown in Figs. 8 and 9:

Figure 8 shows that the depth-based registration approach was successful (models were visually perceived aligned) for the abdominal and skull phantoms, but for the mannequin the reflective surface resulted in a sparse 3D point cloud causing the registration to fail; however, we do not expect such reflective surface on clinical targets. To present a more clinically relevant examples, Fig. 9 shows the model overlaid using depth-based registration on embalmed cadaver feet [22, 23]. This test demonstrated the system’s potential in a realistic surgical setting, showing that the depth-based registration method could align the AR overlay on the cadaver feet.

## Discussion

The evaluation of the AHAT and LT modes of the HoloLens 2’s depth sensor showed a consistent overestimation of the reconstructed depth point clouds along the viewing direction of the camera. For the AHAT mode, the observed mean overestimation over the distance of 50–70 cm was 7.18 ± 4.19 mm, which is comparable to the results reported by Gu et al. [25] for the evaluation of the AHAT mode of the HoloLens 1 (they reported 5.68 ± 2.21 mm overestimation at 70 cm). With the AHAT mode publishing frames at 45 FPS, from our experiment the reconstructed point clouds from the AHAT mode tend to be noisier and with more variability across frames compared to the reconstructed point clouds from the LT mode. This can be observed by the higher standard deviation in distance error for the AHAT mode (see Fig. 4), which also increases when objects are placed further away.

For the LT mode, the reconstructed point clouds had smaller standard deviation (fewer outliers) compared to the point clouds of the AHAT mode. The LT mode, however, showed higher distance mean error compared to the mean error reported by the AHAT for the same distance-to-surface setup. This difference in mean error became smaller for surfaces at 50–70 cm distance from the HL2. The higher mean error at close distances (less than 50 cm) can be due to the far-depth sensing of the LT mode (up to 5 m), where it is mainly used for spatial mapping. Our results suggest that the systematic and more consistent error of overestimation exhibited by the LT mode might be easier to calibrate and correct for compared to the AHAT mode. For this reason, we decided to use the LT mode for the registration experiments.

An important consideration regarding our setup for the depth sensor accuracy experiment is that while we ensured perpendicular alignment of the reference plane to the PV camera’s forward axis, the depth sensor is slightly angulated downward compared to the PV camera, and therefore, it does not maintain a perpendicular view of the target surfaces. However, in practical scenarios, achieving perfect perpendicularity between the depth sensor and all target surfaces is not feasible. Thus, the presented results reflect a representative use case where surfaces are observed from slightly varied angles. Future work could investigate the effect of such angulation on depth estimation accuracy more systematically.

For the evaluation of the registration accuracy of different approaches, we performed 32 registrations with a spine phantom model in various positions. The mean translation errors of the four initialization methods ranged from 12.5 to 17.0 mm, while rotation errors ranged from 0.9 and 1.1 degrees. This result is comparable to the accuracy of depth-based registration methods for the HoloLens reported in previous studies, such as Gsaxner et al. [9], and Haxthauser et al. [8], which is promising, considering that the framework can be further improved. For example, based on the observed systematic overestimation of depth by the HL2 sensor, a calibration method can be investigated to compensate for the error, which can improve the registration accuracy.

The successful registration results obtained for all 32 cases using the two manual registration methods (3P and Manual) indicate their robustness. The automatic registration methods (Eye and Eye RANSAC) did not always achieve accurate registrations. The Eye method had a success rate of only 22%, and successful alignments were achieved only when the initial orientation of the preoperative model was close to the orientation of the intraoperative spine phantom. The additional steps introduced in the Eye RANSAC led to a higher success rate of 34% compared to the Eye method; however, success rate remains relatively low. The relatively low performance of RANSAC may be caused by sub-optimal parameters, as we did not do an extensive RANSAC parameter optimization. This also indicates that accurate initialization of the preoperative model’s rotation remains a challenge for the automatic methods. It is important to note that a direct comparison between Eye RANSAC and Eye’s accuracy cannot be made due to the low success rate of both methods compared to Manual and 3P approaches. Further work could explore ways to increase the success rate of automatic methods, which would facilitate and speed up automatic registration and therefore the usability and clinical applicability. This could include investigating robust global registration algorithms such as TEASER++ [24], which has demonstrated superior performance in robustly handling outliers.

Among the initial alignment methods, the Manual approach demonstrated a robust and efficient way to initialize depth-based registration. Since the user only needed to perform a rough placement of the virtual model onto the physical target, the alignment process was quick and effective, with the subsequent depth-based registration refining the alignment. While the time reported in Table 2 only accounts for the PC processing time, the rough manual placement by the user took approximately 3 s, as shown in the supplementary video. It is important to note that this time is likely to vary depending on the user’s experience and the complexity of the target object. Since the experiment was conducted by a single experienced user and involved one specific target object, the reported times are primarily intended to illustrate the relative differences between the registration methods rather than serve as definitive benchmarks.

Although the HL2 depth-based image-to-patient alignment investigated in this work is not accurate enough for many surgical applications, it has the potential to be improved by calibrating and improving the reconstruction of the intraoperative point cloud. Moreover, further preprocessing and initialization approaches can be investigated for accurate alignment. The current approach relies on the HoloLens’ SLAM after registration, which can suffer from drifting issues. SLAM drift has been reported to be of millimeter to centimeter scale in the HoloLens and therefore remains a significant challenge. In our experiments, we aimed to mitigate the effect of drifting by maintaining a static setup for the depth sensor accuracy experiment and by evaluating the depth registration approaches right after making the alignment for the registration experiment. However, having such controlled experimental setups in real-world scenarios may not be feasible. World locking tools, which stabilize holograms in a stable world-locked coordinate system, can be leveraged to reduce drifting in dynamic clinical environments and ensure accurate alignment throughout the procedure. The drifting issue can also be mitigated by performing recurrent alignments, as the process only takes four seconds, with the first alignment serving to initialize subsequent realignments. Alternatively, recent advancements such as the vision-based drift correction method proposed by Gu et al. [25] can be integrated into the system. Future work should also address the challenge of aligning soft tissue targets, such as the breast, where non-rigid alignment approaches will be necessary.

In comparison with the marker-based alignment approach, marker-less depth-based registration can speed up the alignment step that conventionally requires pinpointing anatomical landmarks using a pointer. By eliminating the need for manual landmark selection, depth-based methods can reduce user errors and minimize sensitivity to outliers.

The depth registration framework proposed in this study is designed to be independent of the target anatomy, offering the potential for broad applicability across different anatomical regions. Figures 8 and 9 show examples of successful depth-based registration on different target objects, while also showing some unsuccessful cases. The framework’s performance can vary based on certain characteristics of the target anatomy, such as surface texture, size, symmetry, and occlusion. Therefore, it is important to further validate the framework for each surgical application. To facilitate the adoption of the method and its wider applicability, we made our method publicly available on GitHub, aiming to promote broader validation and collaborative efforts to improve and adapt the framework for other surgical use cases.

## Conclusion

This study presented a generic marker-less registration framework for the HoloLens 2. The framework uses the depth sensor to reconstruct an integrated point cloud over multiple frames and then uses course and fine point cloud registration approaches to align the preoperative data with the patient and provide a direct overlay of the planning on the target anatomical structure. The framework was evaluated by first assessing the accuracy of the depth sensor of the HoloLens 2 in reconstructing intraoperative point clouds. The quantitative assessment of the depth sensor’s accuracy revealed millimeter-scale mean overestimation error for both the AHAT and LT modes within 50–70 cm distance from the HoloLens. Furthermore, the depth-based registration accuracy of the framework was evaluated with different manual and automatic initialization approaches. The comparison of registration initialization methods using the spine phantom demonstrated the potential of manual approaches to consistently achieve successful alignments. The automatic initial alignment methods showed promise but require improvement, particularly in accurate initialization for subsequent ICP steps. The results show that the registration is in the order of 1–2 cm accuracy and can be achieved within 4 s. This offers a fast and convenient alternative to other time-consuming marker-based registration approaches. Whereas this approach may be sufficient for some procedures, the current alignment accuracy is not sufficient for many other procedures; therefore, further improvements are needed.

## Supplementary material

- GitHub repository containing the framework developed for depth-based registration of 3D preoperative models to intraoperative patient anatomy using the HoloLens 2.
- Video demonstrating depth-based registration using the HoloLens 2 for both automatic and manual initialization approaches.

## Supplementary Information

Below is the link to the electronic supplementary material.

Supplementary file 1 (mp4 85417 KB)
